# Detection of Problems Related to Hormonal Contraceptives in Community Pharmacy: Application of a Structured Questionnaire in Women of Childbearing Age

**DOI:** 10.3390/pharmacy13040112

**Published:** 2025-08-21

**Authors:** Raquel Sicilia-González, Susana Abdala-Kuri, Chaxiraxi Morales-Marrero, Adama Peña-Vera, Alexis Oliva-Martín, Sandra Dévora-Gutiérrez

**Affiliations:** 1 Departamento de Medicina Física y Farmacología, Facultad de Farmacia, Universidad de La Laguna, 38200 Tenerife, Spain; alu0101205878@ull.edu.es (R.S.-G.); extcmoralem@ull.edu.es (C.M.-M.); extapenaver@ull.edu.es (A.P.-V.); sdevora@ull.edu.es (S.D.-G.); 2 Departamento de Ingeniería Química y Tecnología Farmacéutica, Facultad de Farmacia, Universidad de La Laguna, 38200 Tenerife, Spain; amoliva@ull.edu.es

**Keywords:** contraception, community pharmacy, therapeutic adherence, health education, contraception questionnaire

## Abstract

The use of hormonal contraceptives is essential to ensure effective and safe contraception. However, factors such as inadequate prescription, poor adherence, or lack of information can lead to drug-related problems (DRPs) and negative outcomes associated with medication (NOMs). Methods: An observational, descriptive, cross-sectional study was conducted between January and June 2024 in two community pharmacies in Tenerife. It included 316 users of hormonal contraceptives over the age of 18. The main instrument was a structured questionnaire, administered through individual interviews conducted in the Personalized Care Area (PCA). Results: Combined Oral Contraceptives (COCs) were the most frequently used method (72.2%). Adverse reactions were reported by 47.2% of participants, mainly psychiatric disorders (28.1%). Emergency contraception had been used at least once by 43.8% of the respondents. Among COC users, 68.9% reported difficulties with daily adherence, and only 36.7% had adequate knowledge in case of missed doses. Conclusions: Community pharmacists may play a key role in reproductive health by providing personalized counselling, referring patients to other levels of care, and promoting the rational and safe use of hormonal contraceptives.

## 1. Introduction

Access to safe, effective, and personalized contraception is a fundamental pillar of sexual and reproductive health. Globally, more than 60% of women of childbearing age use some form of contraceptive method, although significant differences exist depending on the region, level of development, access to health education, and public health policies [1,2]. These disparities highlight the need for strategies that are better adapted to the sociocultural and clinical context of each population.

In Spain, according to the 2024 survey by the Spanish Society of Contraception, 78.5% of women aged 15 to 49 use some contraceptive method, with male condoms (36.5%) and Combined Oral Contraceptives (COCs) (18%) being the most commonly used [1]. This trend aligns with international surveys, although regional differences persist. According to Bertrand et al., while female sterilization is predominant in Asia, Latin America and North Africa, COCs are the most widespread methods. In sub-Saharan Africa, there has been a progressive shift from oral and injectable contraceptives to long-acting reversible contraceptives (LARCs), such as subcutaneous implants. In regions with a high HIV prevalence, condom use has been promoted as part of public health strategies aimed at preventing its transmission [3].

In the Spanish context, the increased use of COCs has been linked not only to contraceptive purposes but also to therapeutic indications such as menstrual cycle regulation, acne treatment, polycystic ovary syndrome, or endometriosis [4,5]. Their high efficacy is reflected in a very low Pearl Index (0.12–0.34), making them one of the most reliable methods [6]. However, this effectiveness may be compromised by factors such as poor adherence, missed doses, misuse, or lack of health education. These drug-related problems (DRPs), if not identified in time, can lead to negative outcomes associated with medication (NOMs), raising the failure rate to as much as 20% in some cases [7,8].

Discontinuation of treatment is often motivated by adverse reactions—such as breakthrough bleeding, weight gain, or emotional lability—or by a lack of knowledge of how to act in the event of a missed dose [9,10]. Alternatives such as the vaginal ring, transdermal patch, or LARCs (intrauterine devices (IUDs) and subcutaneous implants) have shown improved adherence in certain user profiles [2,4]. Less commonly used methods include progestogen-only pills, quarterly injections, or the diaphragm, while others such as spermicides are virtually obsolete [1,2,3,4,5,6].

When the usual contraceptive method fails, emergency contraception is used. Currently, there are two main options available: ulipristal acetate (30 mg), which is effective up to five days after intercourse and is more effective in overweight women, and levonorgestrel (1.5 mg), whose efficacy decreases after the first 72 h and in women with a high body mass index [11]. Although both have an adequate safety profile [12,13], indiscriminate use may lead to adverse effects such as headaches, nausea, or irregular bleeding, and, in rare cases, ectopic pregnancy or severe reactions such as anaphylaxis [14,15,16]. Repeated use is considered an indirect indicator of poor adherence or inadequate contraceptive planning.

Given the risk profile associated with hormonal contraceptives, especially in women with comorbidities, individualized assessment is essential. Cardiovascular diseases, liver disorders, migraines, obesity, smoking, or drug interactions can significantly increase the risk of severe adverse reactions, such as venous thromboembolism [17,18]. According to the World Health Organization (WHO) medical eligibility criteria, conditions such as hypertension or active smoking in women over 35 years should be considered relative or absolute contraindications [19,20].

In addition, a possible association between COC use and mental health symptoms, particularly in young women, has been described. Although the pathophysiological mechanism is not entirely clear, it is hypothesized that progestogens may interfere with serotonergic neurotransmission. This aspect has gained relevance in recent years and should be considered in clinical practice, especially in adolescents and women with psychiatric histories. Moreover, in the context of personalized medicine, pharmacogenetics applied to contraception is beginning to show clinical utility. Recent studies have identified genetic polymorphisms that may influence the efficacy and tolerability of COCs, opening the door to more individualized prescribing strategies in the future [21,22,23].

However, access to the most effective methods is not always equitable. Differences in education level, income, or geographic location affect access to information and the active prescription of LARCs. This inequality, documented in several recent studies, presents an additional challenge for healthcare professionals involved in contraceptive counselling [22,23].

In this scenario, the role of the community pharmacist is critical. As an accessible healthcare professional with the ability to provide individualized counselling, they can detect DRPs, reinforce adherence, and refer patients to other levels of care when necessary. Their intervention, based on clinical dialogue, helps optimize the rational use of contraceptives and improve health outcomes.

Therefore, the aim of the present study is to design and apply a questionnaire focused on the prescription and dispensing of hormonal contraceptive methods, enabling the identification of the predominant method, assessment of users’ knowledge, detection of DRPs, and description of the sociodemographic profile in a cohort of women of reproductive age, with the ultimate goal of optimizing pharmaceutical practice and contributing to safer, more effective, and better-tailored contraception for each woman.

## 2. Materials and Methods

### 2.1. Study Design and Procedure

An observational, descriptive, and cross-sectional study was conducted between January and June 2024 in two community pharmacies located on the island of Tenerife (San Matías Pharmacy and La Rotonda Pharmacy, in the municipality of Tacoronte), in the province of Santa Cruz de Tenerife. Statistical analysis was performed using R software version 4.5.1., applying descriptive statistics to characterize the study population. Pearson’s Chi-squared test was used to compare qualitative variables between groups. Comparisons were made according to marital status and age group in relation to the indication for contraceptive use, with statistically significant differences observed. Associations were also identified between medical prescription and participants’ age, as well as between therapeutic adherence and the type of contraceptive method used.

The target population consisted of women over the age of 18 who came to collect their hormonal contraceptive method during the study period. Inclusion was consecutive and non-probabilistic, resulting in a convenience sample. In Spain, both oral and non-oral hormonal contraceptives (such as vaginal rings and some subdermal implants) require a medical prescription but are dispensed through community pharmacies. It is a common practice for patients to collect prescribed non-oral contraceptives at the pharmacy after a gynaecological consultation. Therefore, the inclusion of women using non-oral forms is consistent with pharmacy-based data collection. The study protocol was approved by the Research Ethics Committee of the University Hospital of the Canary Islands (code 07/24) and was conducted in accordance with the Declaration of Helsinki and Spanish data protection legislation (Organic Law 3/2018 on the Protection of Personal Data and Guarantee of Digital Rights). All participants signed an informed consent form prior to inclusion in the study. Interviews were conducted in the Personalized Care Area (PCA) of the pharmacy, a space designed to ensure privacy and confidentiality. Each interview lasted approximately 15 to 20 min.

### 2.2. Inclusion and Exclusion Criteria

#### 2.2.1. Inclusion Criteria

Women over 18 years of age;

Current users of a hormonal contraceptive method.

#### 2.2.2. Exclusion Criteria

Refusal to sign the informed consent form;

Exclusive use of non-hormonal contraceptive methods.

#### 2.2.3. Variables Collected

The structured questionnaire, developed ad hoc for this study, collected information on the following:

Sociodemographic characteristics (age, relationship status, and obstetric history);

Contraceptive method used and prescriber;

Reasons for use (contraceptive or non-contraceptive);

Knowledge level regarding the contraceptive method; for example, closed questions in which participants indicate the action they take in the event of a missed dose, or whether they consistently administer the injection at the same time each day;

Treatment adherence;

Presence of adverse reactions or relevant clinical events;

Identification of any drug-related problem (DRP) or negative outcome associated with medication (NOM).

In cases where a DRP or NOM was identified, the community pharmacist provided a specific intervention, consisting of either health education or referral to the primary care physician, depending on the nature of the problem detected.

#### 2.2.4. Instruments and Resources Used

Personalized Care Area (PCA): a confidential space within the pharmacy designed to facilitate clinical interviews and individualized counselling.

Informed Consent Form: a document through which participants voluntarily authorized their inclusion in the study and the processing of their personal data.

“Pharmaceutical Care in Contraception” Questionnaire (Supplementary Materials): a structured questionnaire developed by the research team. No formal validation or pilot testing was conducted; it should therefore be considered an exploratory tool. The questionnaire consisted of a total of 25 closed-ended questions, organized into four sections:

− Sociodemographic profile, 7 questions;
− Pharmacotherapy in contraception, 8 questions;
− Cardiovascular risk factors, 5 questions;
− Treatment adherence: 5 questions.

Bot PLUS^®^ and CIMA^®^: official databases of the General Council of Official Pharmacist Associations and the Spanish Agency of Medicines and Medical Devices, respectively, used for verification and classification of the medications used [22,23].

Google Forms^®^: digital platform used as the electronic case report form for data collection and storage.

Microsoft Excel^®^: software used for the descriptive statistical analysis of the collected variables.

## 3. Results

### 3.1. Sociodemographic Profile

A total of 316 women were included, with a mean age of 28.05 ± 6.10 years, of whom 17.70% were over 35 years old. The most common profile was women with a stable partner (68.99%) and no previous pregnancies (78.20%). The main reason for contraceptive use was pregnancy prevention (41.77%). Among the therapeutic indications, the most frequent were menstrual cycle control (31.01%), polycystic ovary syndrome (16.14%), acne (8.86%), and endometriosis (2.21%). A statistically significant difference was observed between the reason for prescription and marital status, with contraceptive use for pregnancy prevention being more frequent among women with a partner (*p* = 0.01) (Table 1).

The analysis by age group revealed statistically significant differences according to age and the reason for contraceptive use (*p* = 1.746 × 10^−5^). All women over the age of 29 years used contraceptives primarily for pregnancy prevention, whereas among women under 28 years, the predominant indications were menstrual cycle regulation and treatment of polycystic ovary syndrome (Table 2).

### 3.2. Pharmacotherapy in Contraception

The predominant method was COCs (72.20%), followed by the vaginal delivery system (8.50%) and the IUD (5.30%) (Table 3). The prescription of oral pharmaceutical forms showed statistically significant differences depending on the indication (*p* = 2.04 × 10^5^).

A total of 8, 54% reported having experienced iron-deficiency anaemia at some point; among them, 16 were using a copper IUD, although no statistically significant differences were observed (*p* = 0.22) (Table 4). The prescription of an IUD in women with anaemia is contraindicated. The use of a copper IUD may exacerbate this condition. For this reason, the research pharmacist referred those women with anaemia who were prescribed a copper IUD.

Regarding the initial prescription, 69.90% were issued by a gynaecology specialist, 24.15% by a primary care physician, and 1.90% by a midwife, with pregnancy prevention being the main indication in all cases (Table 5). Only 0.63% initiated treatment based on a dermatologist’s recommendation (ages between 21 and 28 years) for dermatological conditions. A total of 2.90% had no medical prescription; among them, seven women were taking COCs for pregnancy prevention and two for menstrual cycle regulation, which led to a referral to primary care.

The most common source of information was family or friends, reported by 196 women, followed by the internet, cited by 65 participants. Only 33 received guidance from a community pharmacist. The remaining respondents obtained information through school-based educational sessions.

### 3.3. Adverse Reactions, Treatment Adherence, and Emergency Contraception

**Figure 1:**
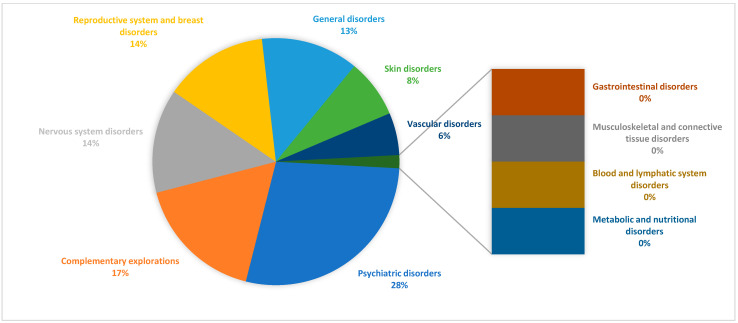
Adverse reactions to contraceptives.

A total of 47.20% of the women reported having experienced at least one adverse reaction associated with their contraceptive method. The most frequent were psychiatric disorders (28.1%), primarily including mood swings, anxiety, and depressive symptoms; general disorders (17.0%), such as headache and fatigue; reproductive system and breast disorders (13.6%), including decreased libido and breast tenderness or enlargement; skin disorders (7.7%), mainly acne and hair loss; and vascular disorders (5.5%), such as fluid retention and peripheral swelling (Figure 1).

**Figure 2:**
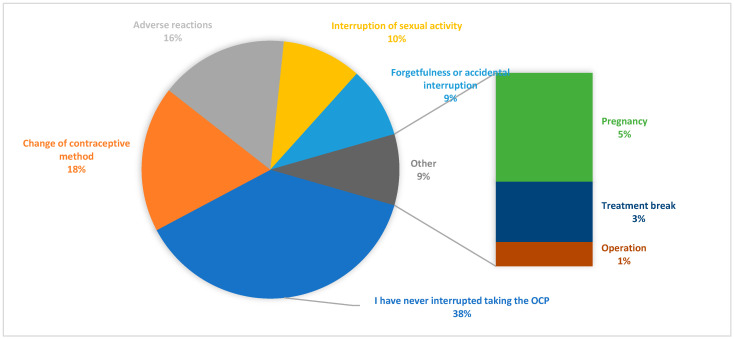
Adherence and interruption of contraceptive treatment: reasons and causes.

Among users of COCs, 68.90% reported difficulties in maintaining daily adherence. For this reason, the research pharmacist offered information about long-acting alternatives, such as the vaginal ring, to optimize therapeutic adherence. In the overall cohort, 37.40% had never interrupted their contraceptive method, 18.10% reported having changed it, 15.90% discontinued use due to adverse reactions, and 8.80% reported forgetfulness or unintentional interruptions (Figure 2).

A total of 43.80% of participants showed poor therapeutic adherence and had used emergency contraception at least once. Among them, 8.80% reported using it one to two times in the past 12 months. One participant reported more than five uses in the past year; this case was recorded as a DRP due to non-adherence, and switching from a COC to a vaginal delivery system was recommended, in addition to referral to primary care. The presence of adverse reactions was associated with therapeutic adherence, with statistically significant differences observed (*p* = 0.02) (Table 6).

Although 86.60% of participants reported being aware of the appropriate action in case of a missed dose, only 36.70% correctly described the procedure. A statistically significant association was observed between therapeutic adherence and the type of contraceptive method used (*p* = 2.7 × 10^−6^) (Table 7).

### 3.4. Chronic Conditions

A total of 21.20% of the participants reported having a chronic condition. Among them, 18.50% suffered from respiratory disorders such as asthma, and 17.10% from endocrine disorders, including hypothyroidism and diabetes mellitus (Table 8). Cardiovascular disorders were present in 11.80% of the sample (hypertension, hypertriglyceridemia, and arrhythmias), while neurological conditions affected 7.90%, with migraine being the most prevalent diagnosis.

### 3.5. Blood Pressure

Only 32.20% of the participants regularly monitored their blood pressure. Among the six women with hypertension, three had controlled levels; the remaining two were referred to primary care to assess antihypertensive treatment. Women who had high blood pressure were provided with health education such as taking blood pressure values routinely, monitoring weight gain, etc., as well as informing their physician of their current use of contraceptives.

In addition, 4.70% of COC users had experienced coagulation disorders, thrombosis, or cardiovascular disease.

### 3.6. Smoking

A total of 13.60% reported being smokers, 14 of whom were over 35 years old. Only one of them was aware of the relative contraindication and had agreed with her physician to continue using COCs.

These women were provided with health information about the contraindication of smoking and taking COCs.

### 3.7. Obesity

Among the participants, 19.62% were overweight and 10.76% were obese. Being overweight and the use of contraceptives are factors that can influence the risk of cardiovascular events. For this reason, overweight women were advised to monitor their weight and maintain it within a healthy range.

### 3.8. Chronic Medication Use

A total of 15.50% of the women were taking at least one medication on a regular basis. Of this group, 38.80% were over the age of 35 years. The most commonly used drugs belonged to group H (thyroid hormone), followed by adrenergic inhalers and antihistamines for systemic use (Table 9). In the studied sample, various therapeutic groups were identified according to the Anatomical Therapeutic Chemical (ATC) Classification and used by the patients. Table 9 presents the distribution of medications by ATC group, highlighting the variety of concomitant treatments alongside contraceptive use. Among the most frequent groups are drugs for the nervous system (N), with a total of 11 women (3.47%), including antidepressants and anxiolytics, and medications for the respiratory system (R), with 13 women (4.1%), mainly inhaled adrenergic and antihistamines. Prescriptions in cardiovascular (C), hormonal (H), and musculoskeletal system (M) groups were also observed.

## 4. Discussion

The results obtained in this study confirm and, in some respects, expand the existing knowledge regarding the use of hormonal contraceptive methods among women of reproductive age.

The most frequent sociodemographic profile—a young woman, in a stable relationship, with no obstetric history— is consistent with the findings of the Spanish Society of Contraception (SEC) survey, reflecting patterns also observed in other national and international studies [1,2]. The most commonly used contraceptive method was the COCs, followed by the vaginal delivery system, which surpassed the IUD. This difference may be explained by the high satisfaction reported by users of the vaginal ring, particularly in terms of convenience, low expulsion rate, and fewer adverse reactions. These features are well documented in the literature, as highlighted in the meta-analysis by Ridgeway et al., supporting its increasing use, especially among women who prioritize autonomy and ease of administration [24].

The lower frequency of IUD use in this cohort is mainly associated with adverse effects related to the copper model, including iron-deficiency anaemia. Previous studies, such as those by Lowe et al. and Godfrey et al., have demonstrated significant decreases in haemoglobin and ferritin levels following prolonged use of this device, supporting the recommendation of routine blood tests in women with heavy menstrual bleeding [25,26]. Similarly, the limited use of the subcutaneous implant and transdermal patch may be explained both by their exclusion from public healthcare funding and by the widespread lack of knowledge about these methods, as highlighted by León-Larios et al. [27]. This situation underscores the need for educational strategies from both community pharmacy services and primary care.

In this study, 16 women used a copper IUD, and statistical analysis showed that there were no significant differences between women using a copper IUD and other contraceptives. The results may be due to a lack of significance or an error due to the small sample size. The relatively small number of users of certain contraceptive methods, particularly copper intrauterine devices (*n* = 16), reduced the statistical power for subgroup comparisons. The lack of significant differences in outcomes such as anaemia among these users may be due to a Type II error.

Access to contraceptives occurred primarily through medical prescription, indicating an appropriate professional approach. However, a small percentage of self-medication (2.9%) reveals the persistence of risky behaviours, since the use of contraceptives without a prior clinical assessment may conceal relevant comorbidities [28,29,30] and lead to NOMs.

Among the non-contraceptive indications, acne treatment stands out as a common practice in young women. This indication is consistent with the findings of Kutlu et al. [30], who observed a higher prevalence of active acne from the age of 20 years, at which point COCs may exert a beneficial effect by reducing sebum production due to their antiandrogenic action. A noteworthy finding was the high frequency of emergency contraception use: 43.8% of participants had used it at least once, and 8,80% more than once in the past year. Although it is a safe and effective resource, repeated use may suggest poor planning, lack of adherence, or insufficient knowledge, increasing hormonal exposure and the risk of adverse reactions [16,31]. The literature consistently highlights the general population’s limited knowledge of emergency contraception, as shown in studies by Jambrina et al. and Nguyen et al. and points out that even when pharmacists possess solid knowledge, it is not always effectively communicated to users [12,13]. In this context, community pharmacies are positioned as a strategic point not only for access but also for providing health education on the correct use of emergency contraception.

The presence of comorbidities and cardiovascular risk factors was significant in the sample, highlighting the need for an individualized assessment prior to prescribing hormonal methods. Conditions such as hypertension, hypothyroidism, obesity, or smoking can alter the benefit–risk balance of COC use. The WHO classifies arterial hypertension as category 3, meaning that the risks generally outweigh the benefits, even when blood pressure levels are controlled [32,33]. Similarly, the combination of COCs with obesity and tobacco use, particularly in women over 35 years of age, has been associated with a substantial increase in the risk of cardiovascular events and is therefore formally contraindicated [32].

Thyroid dysfunction was also detected, with a notable prevalence of hypothyroidism. This condition, which is present in a considerable number of women, requires close endocrinological monitoring, including periodic TSH, T3, and T4 tests, due to potential interactions with contraceptive treatment [34].

Therapeutic adherence, one of the critical factors in the success of contraceptive treatment, showed suboptimal rates. Although 86.6% of participants claimed to know the appropriate course of action in case of a missed dose, only 36.7% answered correctly. This discrepancy between perceived and actual knowledge reveals a gap in health education that must be addressed. The literature has identified age, treatment duration, and the occurrence of adverse events as key factors affecting adherence [8,9,10]. For this reason, long-acting methods such as the vaginal ring and the transdermal patch, or long-acting reversible contraceptives (IUDs or implants), may offer effective alternatives by reducing the risk associated with noncompliance with daily regimens.

Several pharmacist-led interventions were conducted during the study based on the identification of DRPs or NOMs. These included referral to primary care in cases of uncontrolled hypertension, self-medication, cardiovascular history, or repeated emergency contraception use, as well as recommendations for blood tests in women with anaemia using copper IUDs and in patients with chronic treatments. Additionally, personalized counselling was provided on missed pill management, appropriate use of emergency contraception, alternative methods for poor adherence, smoking cessation, and lifestyle modifications.

Beyond physical effects, the potential impact of hormonal contraceptives on mental health has gained increasing attention. Recent studies, such as those by Zettermark et al. and Sultan et al., have suggested an association between COC use and the onset of depressive symptoms or mood disorders, particularly among young women. Although the underlying mechanisms are not fully understood, hormonal modulation of the serotonergic axis has been proposed as a possible explanation. This finding should be taken into account in clinical practice, including emotional screening during initial consultations and follow-up assessments [35,36].

From an equity perspective, considering disparities in access to the most effective contraceptive methods is also necessary. Dehlendorf et al. have documented significant differences in the availability of long-acting contraceptives according to socioeconomic status, while Díaz-Toro et al. have identified disparities based on the educational level of patients in Spain [37,38]. This unequal distribution not only affects method choice but also perpetuates reproductive health inequalities. Therefore, community pharmacists are in a strategic position to act as guarantors of accessibility and equity, providing tailored information, identifying barriers, and facilitating referral to other healthcare levels.

This situation is not unique to the Spanish context. Several European countries, such as France, the United Kingdom, and Germany, have implemented specific programs that assign an active role to community pharmacists in facilitating access to contraceptive methods. In France, since 2022, pharmacists have been authorized to renew prescriptions for oral contraceptives without requiring a new medical evaluation, while in the United Kingdom, the NHS has promoted initiatives allowing community pharmacies to offer contraceptives directly [39,40]. Evaluations of these models have shown significant improvements in treatment continuity, equitable access, and reduced use of emergency contraception. These examples demonstrate that empowering pharmacists in reproductive health is not only feasible but also effective, and they reinforce the need to move forward with its implementation in the Spanish healthcare system.

These findings, along with the results of the present study, highlight the feasibility of introducing practical tools for evaluation and follow-up in community pharmacy settings. The implementation of a structured questionnaire, such as the one used in this study, could be easily integrated into routine community pharmacy practice without requiring significant additional resources. Its systematic use would enable the early detection of medication-related problems, promote adherence, and provide individualized counselling. From a public health policy perspective, its adoption could help reduce the number of unplanned pregnancies, optimize the rational use of hormonal contraceptives, and alleviate the workload in primary care services [40,41]. The most foreseeable barriers would be the lack of time in daily pharmacy practice, the need for specific training in clinical interviewing, and the still-limited integration of community pharmacists into institutional sexual and reproductive health programs [42,43].

Finally, an acknowledgment of the limitations of this study is necessary. Its observational and descriptive design does not allow for causal relationships to be established between the analysed variables. Furthermore, participation was limited to two community pharmacies located in a single geographical region, which may compromise the representativeness of the sample and limit the generalizability of the results to other settings; given that the sample was limited to two community pharmacies in Santa Cruz of Tenerife, the results should not be extrapolated to the population. Further studies across more diverse geographic and socioeconomic settings would be required to assess the generalisability of these findings.

Additionally, the selection of women who actively visited the pharmacy to collect contraceptives may introduce selection bias, as these users might exhibit a higher level of adherence or awareness regarding their treatment compared to the general population. Moreover, the use of self-reported questionnaires may be subject to recall bias or social desirability bias. Nonetheless, data collection through direct interviews conducted in personalized service areas, along with the standardized structure of the questionnaire, helped to partially mitigate these biases.

The discrepancy observed between the perceived and actual knowledge regarding missed dose management illustrates the presence of social desirability and recall bias, which are common in self-reported data. However, this finding also highlights the value of the structured questionnaire in objectively detecting these gaps, allowing pharmacists to target educational interventions accordingly.

Overall, the findings of this study not only reinforce the importance of health education and therapeutic adherence in the use of hormonal contraceptives but also underscore the key role of the community pharmacist as a facilitator of informed, safe, and individualized contraception. Their intervention enables the identification of medication-related problems, the provision of rigorous and personalized information, the optimization of adherence, and referral, when necessary, thus contributing to improved reproductive health outcomes.

Given the exploratory nature of the questionnaire, future studies should include a formal validation process involving expert review for face and content validity, pilot testing in a representative sample to assess feasibility and clarity, and reliability analysis using appropriate statistical methods.

## 5. Conclusions

This questionnaire enables the systematic identification of DRPs, the assessment of users’ knowledge, and the improvement of therapeutic decision-making in hormonal contraception.

The results suggest that community pharmacists may play a relevant role in promoting the rational use of contraceptives, particularly through patient education, early identification of DRPs, and timely referrals to medical care. The findings of this study reinforce the value of structured tools, such as the questionnaire applied here, in community pharmacy practice. This instrument allowed for the identification of DRPs and knowledge gaps and may support pharmacists in improving patient counselling and decision-making related to hormonal contraceptive use.

The findings of this study reinforce the value of structured tools in pharmaceutical practice and highlight the role of community pharmacies as strategic points for optimizing reproductive health and preventing medication-related negative outcomes.

## Figures and Tables

**Table 1:** Sociodemographic profile by marital status and reason for contraceptive use.

Marital Status	Contraception	Therapeutic Indication/Pathology			*p*-Value
With partner	102 (32.27%)	116 (36.70%)	Acne	21 (6.64%)	0.01
			Menstrual cycle regulation	61 (19.30%)	
			Polycystic ovary syndrome	33 (10.44%)	
			Endometriosis	1 (0.31%)	
Without partner	30 (9.49%)	68 (21.51%)	Acne	7 (2.21%)	
			Menstrual cycle regulation	37 (11.70%)	
			Polycystic ovary syndrome	18 (5.69%)	
			Endometriosis	6 (1.89%)	

**Table 2:** Sociodemographic profile by age group and reason for contraceptive use.

Age	Contraception	Pathology			*p*-Value
≤20 years	7 (2.21%)	18 (5.69%)	Acne	3 (0.94%)	0.00001
			Menstrual cycle regulation	10 (3.16%)	
			Polycystic ovary syndrome	4 (1.26%)	
			Endometriosis	1 (0.31%)	
21–28 years (inclusive)	52 (16.45%)	115 (36.39%)	Acne	12 (3.79%)	
			Menstrual cycle regulation	62 (19.62%)	
			Polycystic ovary syndrome	38 (12.02%)	
			Endometriosis	3 (0.94%)	
29–35 years (inclusive)	41 (12.97%)	27 (8.54%)	Acne	11 (3.48%)	
			Menstrual cycle regulation	11 (3.48%)	
			Polycystic ovary syndrome	3 (0.94%)	
			Endometriosis	2 (0.63%)	
≥36 years	32 (10.12%)	24 (7.59%)	Acne	2 (0.63%)	
			Menstrual cycle regulation	15 (4.74%)	
			Polycystic ovary syndrome	6 (1.89%)	
			Endometriosis	1 (0.31%)	

**Table 3:** Pharmaceutical forms and indication for use.

Pharmaceutical Form/Indication	Contraception			Therapeutic Indication/Pathology			*p*-Value
Oral form	78 (24.68%)	COC		150 (47,46%)	Acne	21 (6.64%)	0.00002
					Menstrual cycle regulation	87 (27.53%)	
					Polycystic ovary syndrome	39 (12.34%)	
					Endometriosis	3 (0.94%)	
Non-oral form	54 (17.08%)	IUD	17 (5.37%)	34 (10, 75%)	Acne	6 (1.89%)	
		Vaginal delivery system (vaginal ring)	27 (8.54%)		Menstrual cycle regulation	17 (5.37%)	
		Subcutaneous implant	10 (3.16%)		Polycystic ovary syndrome	8 (2.53%)	
					Endometriosis	3 (0.94%)	

**Table 4:** Presence of anaemia and use of a copper IUD.

Anaemia/IUD Use	With Anaemia Episodes	Without Anaemia Episodes	*p*-Value
Use of an IUD	16 (5.06%)	130 (41.13%)	0.22
Use of a contraceptive method other than an IUD	11 (3.48%)	156 (49.36%)	

**Table 5:** Prescription and/or indication for the contraceptive method.

Prescriber	Indication for the Use of the Contraceptive Method	Total
Dermatologist	Acne	2 (0.63%)
Gynaecology	Acne	8 (2.56%)
	Endometriosis	5 (1.58%)
	Pregnancy prevention	88 (27.84%)
	Menstrual cycle regulation	79 (25.00%)
	Polycystic ovary syndrome	37 (11.70%)
Matron	Pregnancy prevention	5 (1.58%)
	Menstrual cycle regulation	1 (0.31%)
Primary Care Physician	Acne	6 (1.89%)
	Pregnancy prevention	41 (12.97%)
	Menstrual cycle regulation	24 (7.59%)
	Polycystic ovary syndrome	7 (2.21%)
Pharmacist	Pregnancy prevention	2 (0.63%)
None (Self-medication)	Pregnancy prevention	7 (2.21%)
	Menstrual cycle regulation	2 (0.63%)

**Table 6:** Relationship between the presence of adverse reactions and therapeutic adherence.

Adverse Reactions/Therapeutic Adherence	Presence of Adverse Reactions	Lack of Adverse Reactions	*p*-Value
Therapeutic Adherence	93 (29.43%)	82 (25.94%)	0.02
Lack of therapeutic Adherence	56 (17.72%)	85 (26.89%)	

**Table 7:** Therapeutic adherence and pharmaceutical form.

Therapeutic Adherence/Pharmaceutical Form	Oral Pharmaceutical Form (COC)	Other Pharmaceutical Forms			*p*-Value
Therapeutic adherence	137 (43.35%)	75 (22.15%)	IUD	21 (6.64%)	0.00003
			Vaginal Delivery System (vaginal ring)	43 (13.60%)	
			Subcutaneous Implant	11 (3.48%)	
Lack of therapeutic adherence	91 (28.79%)	13 (2.84%)	IUD	6 (1.89%)	
			Vaginal Delivery System (vaginal ring)	6 (1.89%)	
			Subcutaneous Implant	1 (0.31%)	

**Table 8:** Chronic conditions present in the study population.

Physiological System	Pathology	No. of Women (%)
Respiratory system	Asthma	9 (2.84%)
	Allergy	5 (1.58%)
Endocrine system	Hypothyroidism	11 (3.48%)
	Diabetes Mellitus	1 (0.31%)
Cardiovascular system	Arterial hypertension	6 (1.89%)
	Partial obstruction of the right bundle branch	1 (0.31%)
	Arrhythmias	1 (0.31%)
	Hypertriglyceridemia	1 (0.31%)
	Long QT syndrome	1 (0.31%)
Blood and lymphatic system	“Clotting disorders”	1 (0.31%)
	Anaemia	2 (0.63%)
	Haemophilia	1 (0.31%)
	Factor V Leiden syndrome	1 (0.31%)
	Thalassaemia	2 (0.63%)
	Raynaud’s phenomenon	1 (0.31%)
Nervous system	Migraine	3 (0.94%)
	Multiple sclerosis	1 (0.31%)
	Glaucoma	2 (0.63%)
Psychiatric	Anxiety and depression	4 (1.26%)
Digestive system	Irritable bowel syndrome	2 (0.63%)
	Nonspecific terminal ileitis	1 (0.31%)
Immune system	Systemic lupus erythematosus	2 (0.63%)
	Myasthenia gravis	1 (0.31%)
Skin and subcutaneous tissue	Psoriasis	1 (0.31%)
	Alopecia	1 (0.31%)

**Table 9:** Classification of medications prescribed to the study population.

ATC Group	Classification	Nº of Women
A: Alimentary tract and metabolism	A02B: Drugs for peptic ulcer and gastroesophageal reflux disease	1 (0.31%)
	A03A: Drugs for functional gastrointestinal disorders	1 (0.31%)
	A03B: Belladonna and derivatives	1 (0.31%)
	A04A: Antiemetics and antinauseants	1 (0.31%)
	A10B: Blood glucose-lowering drugs, excluding insulins	1 (0.31%)
	A12A: Calcium	1 (0.31%)
B: Blood and blood-forming organs	B01A: Antithrombotic agents	1 (0.31%)
	B03A: Iron preparations	2 (0.63%)
C: Cardiovascular system	C02A: Centrally acting antiadrenergic agents	4 (1.26%)
	C09A: ACE inhibitors	1 (0.31%)
	C09C: Angiotensin II antagonists	1 (0.31%)
	C10A: Lipid-modifying agents	1 (0.31%)
D: Dermatological	D07A: Topical corticosteroids	1 (0.31%)
	D11A: Other dermatological preparations	1 (0.31%)
G: Genitourinary system and sex hormones	G03D: Progestogens	1 (0.31%)
H: Systemic hormonal preparations, excluding sex hormones and insulins	H02A: Corticosteroids for systemic use	3 (0.94%)
	H03A: Thyroid hormones	10 (3.16%)
L: Antineoplastic and immunomodulatory agents	L01B: Antimetabolites	2 (0.63%)
	L04A: Immunosuppressants	1 (0.31%)
M: Musculoskeletal system	M01A: Non-steroidal anti-inflammatory and antirheumatic drugs (NSAIDs)	3 (0.94%)
N: Nervous system	N02A: Opioids	2 (0.63%)
	N02B: Other analgesics and antipyretics	1 (0.31%)
	N02C: Antimigraine preparations	2 (0.63%)
	N03A: Antiepileptics	1 (0.31%)
	N05B: Anxiolytics	1 (0.31%)
	N05C: Hypnotics and sedatives	1 (0.31%)
	N06A: Antidepressants	4 (1.26%)
	N07A: Parasympathomimetic	1 (0.31%)
P: Antiparasitic products, insecticides, and repellents	P01B: Antimalarials	2 (0.63%)
R: Respiratory system	R03A: Adrenergic, inhalants	7 (2.21%)
	R06A: Antihistamines for systemic use	6 (1.89%)
S: Sensitive organs	S01E: Antiglaucoma preparations and miotics	1 (0.31%)

## Data Availability

The study data are not available because the repository designated for their storage had a limited duration, which has now expired. Once this period concluded, in accordance with the established preservation conditions, access to and availability of the information ceased to be in effect.

## Supplementary Materials

The following supporting information can be downloaded at: https://www.mdpi.com/article/10.3390/pharmacy13040112/s1, “Pharmaceutical Care in Contraception” Questionnaire.

## Abbreviations

COC	Combined Oral Contraceptive
LARC	Long-Acting Reversible Contraceptive
DRP	Drug-Related Problem
NOM	Negative Outcome associated with Medication
IUD	Intrauterine Device
PCA	Personalized Care Area
WHO	World Health Organization
ATC	Anatomical Therapeutic Chemical classification
