# Supplementary material for: Detection of Problems Related to Hormonal Contraceptives in Community Pharmacy: Application of a Structured Questionnaire in Women of Childbearing Age
Source: Pharmacy (Basel). 2025 Aug 21;13(4):112. doi: 10.3390/pharmacy13040112 (PMC12389131; doi:10.3390/pharmacy13040112)
Supplement: Supplementary file 1 [file pharmacy-13-00112-s001.zip › pharmacy-3790430-supplementary.pdf]

Annex 1: **PHARMACEUTICAL CARE INTERVIEW ON CONTRACEPTION**

Sociodemographic Profile

1. **Age:**

   ☐

2. **Marital status:**

   ☐ Single

   ☐ In a relationship

3. **Have you ever had a previous pregnancy?**

   ☐ Yes

   ☐ No

4. **How long have you been using the contraceptive method?**

   ☐ 1 year or less

   ☐ More than 1 year, but less than 5 years

   ☐ 5 years or more

5. **Please indicate:**

   Weight: _____ kg

   Height: _____ cm

6. **Do you have a family history of breast cancer?**

   ☐ Yes

   ☐ No

7. **Have you ever been diagnosed with anemia?**

   ☐ Yes

   ☐ No

Pharmacotherapy in Contraception

8. **Which contraceptive method are you currently using?**

   ☐ Condom

   ☐ Oral contraceptive pill (OCP)

   ☐ Vaginal ring

   ☐ Patch

   ☐ Implant

   ☐ Intrauterine device (IUD)

   ☐ Spermicides

   ☐ Other (specify): ___________

9. **Who prescribed the contraceptive method you are currently using?**

☐ Primary care physician

☐ Gynecology specialist

☐ Other healthcare professional (specify)

☐ None of the above, self-medication

10. **If you use the contraceptive method without a prescription (self-medication), how did you obtain the information?**

☐ Friends/relatives

☐ Internet

☐ Educational institution (school, institute, university, etc.)

☐ Other (specify): __________

11. **Why did you start using the contraceptive method?**

☐ To prevent pregnancy

☐ Better menstrual cycle control

☐ Acne

☐ Polycystic ovary syndrome

☐ Other (specify): __________

12. **Are you currently taking any other medication?**

☐ No, I am not taking any other medication

☐ Yes, I am taking other medication

13. **Have you experienced any side effects while using your current contraceptive method?**

☐ No, I have not experienced any side effects

☐ Yes, I have experienced side effects, and they were: __________

14. **How many times have you used an emergency oral contraceptive (morning-after pill)?**

☐ 0 times

☐ 1–3 times

☐ 4–6 times

☐ More than 6 times

15. **In the past 12 months, how often have you used the morning-after pill?**

☐ Never

☐ Occasionally (1–2 times)

☐ Regularly (3–5 times)

☐ Frequently (more than 5 times)

Cardiovascular Risk

16. **Do you have any diagnosed medical condition?**

☐ Yes, I have __________

☐ No, I do not have any diagnosed medical condition

17. **Are you currently taking any medication to treat this condition?**

☐ Yes, I take __________

☐ No, I do not take any medication

18. **Since starting contraceptive treatment, have you experienced coagulation problems, thrombosis, or cardiovascular diseases?**

☐ Yes

☐ No

19. **Do you have a family history of cardiovascular disease?**

☐ Yes

☐ No

20. **Do you smoke?**

☐ Yes

☐ No

Treatment Adherence

21. **Do you often forget to take your medication?**

☐ Yes

☐ No

22. **Do you know what to do in case of a missed dose?**

☐ Yes

☐ No

23. **If you forget to take an oral contraceptive pill (OCP), what action would you take?**

☐ I take the pill as soon as I remember, even if it means taking two pills at the same time if I have only missed one dose

☐ I do not take it and skip the missed dose

24. **If you have ever interrupted the use of OCPs, what was the reason?**

☐ I have never interrupted OCP use

☐ Change of contraceptive method

☐ Temporary discontinuation of sexual activity

☐ Side effects

☐ Forgetfulness or accidental interruption

☐ Other (specify): __________

25. **Do you find it difficult to take your medication at the same time every day?**

☐ Yes

☐ No

Thank you for your cooperation!
